# Individual socio-demographic factors and perceptions of the environment as determinants of inequalities in adolescent physical and psychological health: the Olympic Regeneration in East London (ORiEL) study

**DOI:** 10.1186/s12889-015-1459-1

**Published:** 2015-02-15

**Authors:** Neil R Smith, Daniel J Lewis, Amanda Fahy, Sandra Eldridge, Stephanie JC Taylor, Derek G Moore, Charlotte Clark, Stephen A Stansfeld, Steven Cummins

**Affiliations:** Centre for Psychiatry, Barts and The London School of Medicine and Dentistry, Queen Mary University of London, Old Anatomy Building, Charterhouse Square, London, EC1M 6BQ, UK; Department of Social and Environmental Health Research, London School of Hygiene and Tropical Medicine, 15-17 Tavistock Place, London, WC1H 9SH, UK; Centre for Primary Care and Public Health, Barts and The London School of Medicine and Dentistry, Queen Mary University of London, 4 Newark Street, London, E1 2AT, UK; Institute for Research in Child Development, School of Psychology, University of East London, Stratford Campus, Water Lane Stratford, London, E15 4NO, UK

**Keywords:** Public health, Adolescent, Health inequalities, East London, Social determinants, Neighbourhood, Mental health, Physical activity, Self-rated general health, Longstanding illness

## Abstract

**Background:**

Populations living in urban areas experience greater health inequalities as well as higher absolute burdens of illness. It is well-established that a range of social and environmental factors determine these differences. Less is known about the relative importance of these factors in determining adolescent health within a super diverse urban context.

**Methods:**

A cross-sectional sample of 3,105 adolescent participants aged 11 to 12 were recruited from 25 schools in the London boroughs of Newham, Tower Hamlets, Hackney and Barking & Dagenham. Participants completed a pseudo-anonymised paper-based questionnaire incorporating: the Warwick-Edinburgh Mental Well-being Scale used for assessing positive mental well-being, the Short Moods and Feelings Questionnaire based on the DSM III-R criteria for assessment of depressive symptoms, the Youth-Physical Activity Questionnaire and a self-assessment of general health and longstanding illness. Prevalence estimates and unadjusted linear models estimate the extent to which positive well-being scores and time spent in physical/sedentary activity vary by socio-demographic and environmental indicators. Logistic regression estimated the unadjusted odds of having fair/(very)poor general health, a long standing illness, or depressive symptoms. Fully adjusted mixed effects models accounted for clustering within schools and for all socio-demographic and environmental indicators.

**Results:**

Compared to boys, girls had significantly lower mental well-being and higher rates of depressive symptoms, reported fewer hours physically active and more hours sedentary, and had poorer general health after full adjustment. Positive mental well-being was significantly and positively associated with family affluence but the overall relationship between mental health and socioeconomic factors was weak. Mental health advantage increased as positive perceptions of the neighbourhood safety, aesthetics, walkability and services increased. Prevalence of poor health varied by ethnic group, particularly for depressive symptoms, general health and longstanding illness suggesting differences in the distribution of the determinants of health across ethnic groups.

**Conclusions:**

During adolescence perceptions of the urban physical environment, along with the social and economic characteristics of their household, are important factors in explaining patterns of health inequality.

## Background

Urban areas have been repeatedly associated with greater health inequalities as well as higher absolute burdens of illness [1,2]. The determinants of health in urban areas and neighbourhoods have been well characterised and are centred upon the context of the built and physical environment and the composition of individuals and their socioeconomic environment [3,4]. Environmental factors promoting health include high quality public infrastructure and housing, access to healthy food, services and businesses, green or blue spaces, and low pollution with a favourable climate or geography. Socioeconomic factors associated with poorer adult health include unemployment, lower quality employment, working or living conditions, education, health and social services, and low levels of community participation, cohesion and safety [5]. Urban areas that include neighbourhoods which concentrate socioeconomically and environmentally deprived populations [6], result in specific and localised patterns of inequality and consequent ill health.

However, in the UK, much of the information about ill health and its underlying determinants has been captured by national surveys employing sampling strategies which commonly exclude adolescents [7-10]. Adolescent health profiles which do exist tend to be ecological in perspective, broadly descriptive and unable to explicitly examine outcomes in relation to wider determinants at an individual level [11]. So, while it is well-established that a multi-dimensional range of both social and environmental factors determine adult population health [5], less is known about the relative importance of each of these factors in determining health during adolescence.

It is also important to consider whether such elements underpinning health inequalities operate similarly within adolescents additionally exposed to super-diverse urban contexts. For example, the prevalence of childhood obesity at age 10/11 years in East London, UK, is significantly higher than the national average whereas smoking rates are lower [12]. Given the considerable evidence linking social and environmental exposures in childhood to later life outcomes [13], these inequalities and their determinants may continue into adulthood. Indeed, the prevalence of adult common mental disorder assessed by a London-based community health study was nearly twice that of the national survey estimate. Meanwhile the same study identified significantly lower prevalence within the urban context of health behaviours such as hazardous alcohol drinking [14]. Therefore such urban-specific patterns of ill health require further investigation as the individual and environmental predictors of adolescent health inequalities could potentially identify key determinants operating prior to adulthood. Identification of early life risk factors may also suggest a means for early prevention and alleviation of later life inequalities.

Here we present the cross-sectional baseline survey of adolescents participating in the longitudinal Olympic Regeneration in East London (ORiEL) cohort study. We explore associations between demographic, socioeconomic and environmental factors with physical/sedentary activity, physical health and psychological well-being. This assessment of the determinants of health inequalities in East London is especially timely given the neighbourhood has undergone rapid and large scale socio-demographic and physical environment transformations in recent years primarily due to the 2012 Olympic Games-led regeneration programme. Moreover, East London is one of the most under-enumerated areas of England and Wales according to the 2011 census [15] and remains relatively uncharted over the past decade in terms of its population characteristics [16] and especially of peoples’ environments [17] and their consequent health.

## Methods

### Study design and participants

The ORiEL study is a prospective cohort study aimed at assessing the impact of urban regeneration on the health of young people and their families. The full study protocol is published elsewhere [18]. Briefly, the cross-sectional baseline survey presented here comprises 3,105 adolescents in year 7 of secondary school (aged 11–12 years) who completed a paper-based questionnaire during the 6 months (January to July 2012) prior to the start of the London 2012 Olympic and Paralympic Games. Special needs-schools and pupil referral units were excluded from the sample frame. Respondents were recruited from six schools in each of the London boroughs of Newham, Hackney and Barking and Dagenham and from seven schools in Tower Hamlets. These boroughs are characterised by higher levels of social, economic and environmental deprivation than the English and the London average [19], as well as being highly ethnically diverse with around two thirds of residents self-identifying with an ethnic minority group at the 2011 Census [20]. Consequently 80% of the sample is from an ethnic minority.

Schools were selected using simple randomisation within each borough with refusals replaced by eligible schools from the same borough. The most common reason for schools refusal was “research fatigue” with teachers being repeatedly approached by external agencies to participate in research projects. This suggests that personal preferences of organising staff were a cause of refusal rather than pupil characteristics. As the survey was undertaken in school settings during school hours it is expected that this will minimise response bias compared to population-based survey methods. To attain sample power the whole school year was surveyed in seven schools which had relatively small year groups. The remaining 18 larger schools provided an allocation of adolescents selected on the basis of school timetabling logistics. Adolescents were not streamed by academic abilities as the survey was carried out during PE lessons. Despite the refusals the consequent sample featured single sex schools and drew on the largest and smallest schools in the four boroughs which were affiliated to a range of religious denominations. A comparison with census data will assess whether the sample was unrepresentative of the local total population of a similar age.

### Measures

The ORiEL paper questionnaire was based on validated instruments deployed to assess a range of outcomes.

### Health outcomes

Mental well-being was assessed using the Warwick-Edinburgh Mental Well-being Scale (WEMWBS) [21]. This is a positively-worded 14 point scale with five response categories capturing eudaimonic and hedonic perspectives of positive mental health. The total score ranges between 14 (lowest well-being) and 70 (highest) and is reported as a mean value within groups. It has been validated in adolescents [22] and cross culturally [23] and was introduced as a core module to the nationally representative Health Survey for England in 2010 [7]. Depressive symptoms were investigated using the Short Moods and Feelings Questionnaire [24]. This is a validated 13 item short form of the 32 item Moods and Feelings Questionnaire scored on a three point scale between “true”, “sometimes true” and “not true”. Scores range between 0 and 26 with total score of 8 or more indicating depressive symptoms. Physical and sedentary activity was estimated by the self-reported Youth-Physical Activity Questionnaire (Y-PAQ) [25]. This questionnaire assesses the accumulated time spent physically active or sedentary respectively over the previous seven days outside of school. The total time spent physically active in recreational games and sports outside of school was derived. Conversely the total time involved in sedentary activities, including screen time, was also estimated for outside of school. Individuals reporting >75 hours total activity per week (outside of school) were excluded from the analysis due to likely over-reporting of time. Participants were asked to rate their own health in general and responses were dichotomised to fair/poor/very poor as opposed to good/very good [26]. Longstanding illness or disability was defined as having a health problem that has troubled the participant over a period of time, or likely to affect the participant over a period of time [27]. Examples included asthma, anaemia, eczema, diabetes, epilepsy, hearing and eyesight problems and Chronic Fatigue Syndrome.

### Exposures

The distribution of health outcomes was explored across a range of individual demographic and household socioeconomic indicators as well as by individual perceptions of the local environment.

### Demographic factors

Demographic indicators included borough of residence, gender, ethnicity and whether the respondent was born in the UK. Self-reported ethnicity used the wording and adapted categories of the England and Wales Census 2011 [28]. These sample-specific and age appropriate categories were derived via extensive piloting to capture the characteristics of the highly ethnically diverse sample in East London. The analysis includes the eight largest groups in the study, namely: White UK, White Mixed (‘White UK and any other background’) and Indian, Pakistani, Bangladeshi, Black Caribbean, Black African. All other ethnic minority groups collapsed to the Other category for analysis by health outcome.

### Socioeconomic factors

Adolescents were asked whether their parents/carers “had a job” to determine whether both parents were not in paid employment (unemployed), if one parent was not in paid employment (one employed), both were in paid employment (both employed), or whether they were a lone parent in paid employment (lone parent employed/not employed). Household socioeconomic circumstances were quantified by the Family Affluence Scale [29]. This four item scale has been validated in young people cross-nationally [29] and is predictive of physical activity and self-reported general and mental health [30]. Adolescents were additionally asked whether they were in receipt of means-tested free school meals.

### Environmental factors

Adolescents were asked for perception of their local neighbourhood, defined as the area they could walk to within 15 minutes from their house, using selected domains from an adapted and age-appropriate ALPHA (Assessing Levels of Physical Activity and Fitness) questionnaire [31]. Statements about perceptions of neighbourhood safety, aesthetics and walkability/cycleability were rated on a four point scale (strongly agree to strongly disagree) with an additional domain asking how near in minutes participants lived to a range of businesses or services. Due to a positively skewed distribution of the summed scores, all four domains were split into tertiles representing a relatively positive, mixed or negative perception of each environmental characteristic.

### Statistical analysis

Analyses were completed using Stata 13.1 (Stata Corporation, Texas, USA). There are four stages to the analysis. The first stage uses the total sample available for each outcome to estimate the unadjusted mean mental well-being total score, mean total time spent in physical/sedentary activity, and the proportion self-reporting fair/(very) poor general health, longstanding illness and depressive symptoms for all participating adolescents across the range of demographic, socioeconomic and environmental indicators. An unpaired *t*-test (for mean outcomes) and logistic regression (for binary outcomes) was used to test for significant differences between sub-categories of covariates. The second stage repeated this analysis using a complete case sample for each outcome. Third, the prevalence of each outcome was then fully adjusted for all demographic, socioeconomic and environmental factors using a complete case mixed effects linear and logit regression models to account for clustering at school level. Likelihood ratio tests were used to assess whether the variance for each outcome was attributable to the clustering effect within schools. Lastly, the relationship between all health outcomes was examined using mixed effects logistic and linear regression to account for clustering, adjusted for gender, country of birth, ethnicity, borough, parental employment, family affluence, and all neighbourhood characteristics.

### Ethical approval

The study has approval from Queen Mary University of London Ethics Committee (QMREC2011/40), the Association of Directors of Children’s Services (RGE110927) and the London Boroughs Research Governance Framework (CERGF113).

### Consent

Participant address details could not be released by the schools. Therefore, one week prior to survey, the school provided each adolescent with the age-appropriate study information sheet and a study information sheet to take home to their parent/carer. The parental letter presented the opportunity to actively opt the adolescent out of the study. Therefore parental consent was passively obtained if the opt-out form was not returned by the child. During the survey visit the questionnaire was explained orally immediately prior to completion, all adolescents additionally provided active written assent prior to completing the survey, and all adolescents were reminded that they were free to withdraw at any time without consequence. Immediately following survey completion all students were provided with a copy of their assent form and a duplicate of the age-appropriate information sheet. They were invited to contact the ORiEL project if they had further questions.

## Results

Table 1 shows that the socio-demographic characteristics of ORiEL baseline sample were broadly similar to a cohort of similar ages observed at the most recent 2011 Census with some exceptions. The ORiEL sample was slightly under-represented by females and Bangladeshi and White UK respondents; this ethnic difference contrasted with an ORiEL over-sample of White Other and Mixed White ethnic groups. The high proportion of White Other groups included recent migrants from European Union states and will have contributed significantly to the higher than expected numbers of participants born overseas. Overall, the response rate was 87% and the study sample (N = 3,105 in school year 7) can be estimated at approximately 25% of the entire age group attending state schools in the catchment areas (N = 12,136, in school year 6).

**Table 1:** **Demographic comparisons of ORiEL adolescent sample with UK census information**

	**ORiEL study sample at 2012 baseline**		**2011 census in ORiEL catchment area** ^**a**^	
	**N**	**%**	**N**	**%**
**Gender** ^**b**^				
Male	1756	56.6	6205	50.9
**Ethnic group** ^**c**^				
White: UK	598	19.5	13328	24.0
White: Other	399	13.0	4454	7.4
White: Mixed	380	12.4	4648	7.7
Asian: Indian	108	3.5	2846	4.2
Asian: Pakistani	130	4.2	2888	4.1
Asian: Bangladeshi	508	16.6	12976	22.4
Asian: Other	27	0.9	1943	3.0
Black: Caribbean	147	4.8	2772	4.6
Black: African	364	11.9	8666	14.3
Black: Other	242	7.9	2511	4.2
Other	163	5.3	2392	4.0
**Nativity** ^**c**^				
Born overseas	628	20.7	26697	12.2
**Borough** ^**b**^				
Newham	895	28.8	3,967	32.7
Tower Hamlets	807	26.0	2,771	22.8
Barking & Dagenham	670	21.6	2,559	21.1
Hackney	733	23.6	2,839	23.4
**Economic Activity** ^**d**^				
Both unemployed	279	10.4	23536	11.7
One parent employed	941	35.07	67187	33.4
Both parents employed	1054	39.28	61638	30.6
Lone parent employed	235	8.76	23145	11.5
Lone parent unemployed	174	6.49	25917	12.9

^a^In order to protect against disclosure of personal information age groups have been combined and some records have been swapped between different geographic areas.

^b^Census sample is age 10 at March 2011.

^c^Census sample is age 10–14 at March 2011.

^d^Census sample is all parents aged 16 and over with dependent children at March 2011.

The following Tables 2, 3, 4, 5, 6 and 7 present observations based on the analytic complete case sample. The total sample available for analysis is also shown in each table and demonstrates that differences in prevalence and trends across categories of covariates did not differ greatly between the total sample available for analysis and the complete case sample used to fully account for demographic, socioeconomic and environmental factors.

**Table 2:** **Mean mental well**-**being scores** (**WEMWBS** ^**e**^) **by selected demographic**, **socioeconomic and environmental factors**

	**Full sample** **(N)** **Unadjusted mean** ^**c**^	**Analytic sample** **(N** **=** **1689)** **Unadjusted mean**	**Analytic sample** **(N** **=** **1689)** **Fully adjusted mean** **(95** **%** **CI)** ^**d**^	
**Demographic factors**				
**Gender**				
†Male	51.6 (1692)	52.3 (898)	52.3	[51.7,52.9]
Female	50.5 (1293)***	50.8 (791)***	50.8***	[50.2,51.4]
**Ethnic group**				
†White: UK	51.0 (579)	51.4 (352)	51.3	[50.4,52.3]
White: Mixed	50.9 (362)	51.3 (183)	51.4	[50.1,52.6]
Asian: Indian	51.6 (106)	52.6 (71)	52.5	[50.5,54.5]
Asian: Pakistani	49.8 (125)	49.9 (78)	50.4	[48.5,52.4]
Asian: Bangladeshi	50.9 (500)	51.5 (335)	51.3	[50.3,52.3]
Black: Caribbean	52.6 (138)	52.6 (65)	52.7	[50.6,54.8]
Black: African	52.0 (342)	51.8 (172)	51.7	[50.4,53.0]
Other	51.1 (803)	51.9 (433)	52.0	[51.1,52.8]
**Nativity**				
†UK Born	51.1 (2344)	51.6 (1376)	51.6	[51.2,52.1]
Born overseas	51.3 (595)	51.6 (313)	51.5	[50.5,52.5]
**Borough**				
†Newham	50.3 (856)	50.7 (421)	50.8	[50.0,51.6]
Tower Hamlets	51.7 (790)**	51.9 (476)*	51.9	[51.1,52.7]
Barking & Dagenham	51.7 (641)**	52.1 (414)*	52.5*	[51.6,53.3]
Hackney	51.1 (698)	51.5 (378)	51.1	[50.2,52.0]
**Socioeconomic factors**				
**Parental economic activity**				
†Both unemployed	50.7 (273)	50.8 (185)	51.0	[49.6,52.4]
One parent employed	51.1 (920)	51.7 (580)	51.9	[51.2,52.6]
Both parents employed	51.7 (1020)	51.9 (665)	51.5	[50.7,52.2]
Lone parent employed	50.4 (229)	50.6 (144)	50.7	[49.3,52.2]
Lone parent unemployed	52.0 (171)	52.3 (100)	53.2*	[51.5,55.0]
Doesn’t live with parents	47.6 (28)	49.3 (15)	49.5	[45.0,54.0]
**Family affluence** ^**a**^				
†Low	50.2 (302)	50.1 (179)	50.0	[48.8,51.3]
Moderate	50.8 (1527)	51.3 (906)	51.3*	[50.8,51.9]
High	51.9 (1034)**	52.5 (604)*	52.5**	[51.8,53.2]
**Free school meals**				
†No meals	51.3 (1758)	51.7 (1100)	51.6	[51.0,52.1]
Receives free meals	50.8 (1173)	51.4 (589)	51.6	[50.8,52.4]
**Environmental factors**				
**Neighbourhood safety** ^**b**^				
†Safe	52.8 (619)	53.3 (456)	52.4	[51.6,53.2]
Mixed	51.7 (762)*	51.8 (573) *	51.7	[51.0,52.4]
Not safe	50.0 (942) ***	50.3 (660) ***	50.9*	[50.2,51.6]
**Neighbourhood aesthetics** ^**b**^				
†Pleasant	53.8 (554)	53.8 (439)	53.6	[52.7,54.4]
Mixed	51.7 (676)***	52.0 (508)***	51.9**	[51.2,52.7]
Unpleasant	49.8 (1050)***	50.0 (742)***	50.2**	[49.5,50.8]
**Neighbourhood walk**-**cycleability** ^**b**^				
†Easy to walk/cycle	52.8 (478)	53.1 (367)	53.2	[52.3,54.1]
Mixed	50.8 (616)***	51.0 (487)***	51.2**	[50.5,52.0]
Not easy to walk/cycle	51.3 (1067)***	51.3 (835)**	51.1**	[50.5,51.7]
**Proximity to businesses & services** ^**b**^				
†Close by	52.8 (626)	53.0 (480)	52.7	[52.0,53.5]
Mixed	51.6 (809)*	51.9 (581)*	52.0	[51.3,52.7]
Far away	50.4 (890)***	50.2 (628)***	50.3**	[49.7,51.0]
Likelihood ratio test v linear regression			p = 1.00	

†Reference category.

*p < 0.05; **p < 0.01; ***p < 0.001.

^a^0 to 2 items = low score; 3 to 5 items = moderate score; 6 to 9 items = high score.

^b^Individual items were summed were summed for each scale and split into tertiles owing to the skewed distribution.

^c^Full sample N varies by each outcome due to missing data.

^d^Adjusted for all demographic, socioeconomic and environmental indicators accounting for clustering within schools.

^e^Maximum well-being score = 70.

**Table 3:** **Prevalence estimates and odds ratios for symptoms of depression on the Short Moods and Feelings Questionnaire** (**SMFQ** > =**8**) **by selected demographic**, **socioeconomic and environmental factors**

	**Full sample** **(N)** **Prevalence** **%** ^**c**^	**Analytic sample** **(N** **=** **1641)** **Prevalence** **%**	**Analytic sample** **(N** **=** **1641)** **Fully adjusted odds ratio** **(95** **%** **CI)** ^**d**^	
**Demographic factors**				
**Gender**				
†Male	18.4 (1584)	16.2 (872)	1.00	-
Female	27.5 (1237)***	27.4 (769)***	2.06***	[1.60,2.65]
**Ethnic group**				
†White: UK	24.8 (552)	25.2 (345)	1.00	-
White: Mixed	24.2 (343)	25 (180)	1.04	[0.67,1.63]
Asian: Indian	15.8 (101)	11.8 (68)*	0.41*	[0.18,0.91]
Asian: Pakistani	24.6 (122)	23.7 (76)	0.82	[0.44,1.53]
Asian: Bangladeshi	18.5 (487)*	17.5 (326)*	0.66	[0.43,1.03]
Black: Caribbean	20.7 (135)	24.2 (66)	1.07	[0.56,2.05]
Black: African	21.1 (313)	19.1 (162)	0.79	[0.48,1.29]
Other	23.8 (741)	21.5 (418)	0.88	[0.61,1.27]
**Nativity**				
†UK Born	22.3 (2222)	21.7 (1337)	1.00	-
Born overseas	22.2 (554)	20.4 (304)	1.02	[0.73,1.45]
**Borough**				
†Newham	24.5 (795)	22.9 (406)	1.00	-
Tower Hamlets	19.3 (751)*	19.3 (466)	0.78	[0.54,1.14]
Barking & Dagenham	24.4 (607)	23 (400)	0.91	[0.62,1.34]
Hackney	21.6 (668)	20.9 (369)	0.80	[0.54,1.19]
**Socioeconomic factors**				
**Parental economic activity**				
†Both unemployed	25.6 (262)	23.1 (182)	1.00	-
One parent employed	21.5 (871)	21.8 (559)	0.84	[0.53,1.33]
Both parents employed	21.2 (970)	20.4 (652)	0.72	[0.43,1.20]
Lone parent employed	25.3 (217)	22.3 (139)	0.71	[0.39,1.31]
Lone parent unemployed	20.9 (163)	18.8 (96)	0.52	[0.27,1.00]
Doesn’t live with parent	29.6 (27)	46.2 (13)	2.23	[0.67,7.41]
**Family affluence** ^**a**^				
†Low	25.3 (273)	25.5 (165)	1.00	-
Moderate	22.4 (1459)	21.6 (885)	0.83	[0.55,1.24]
High	21.4 (967)	20.1 (591)	0.73	[0.47,1.13]
**Free school meals**				
†No meals	21.6 (1667)	21.3 (1074)	1.00	-
Receives free meals	23.3 (1106)	21.7 (567)	0.91	[0.66,1.25]
**Environmental factors**				
**Neighbourhood safety** ^**b**^				
†Safe	16.1 (597)	15.5 (446)	1.00	-
Mixed	19.7 (731)	18.9 (556)	1.06	[0.75,1.51]
Not safe	29.3 (895)***	27.9 (639)***	1.53*	[1.08,2.17]
**Neighbourhood aesthetics** ^**b**^				
†Pleasant	15.8 (537)	13.8 (427)	1.00	-
Mixed	19.5 (647)	19.3 (493)*	1.41	[0.97,2.05]
Unpleasant	28.2 (997)***	27.5 (721)***	2.09***	[1.46,2.99]
**Neighbourhood walk**-**cycleability** ^**b**^				
†Easy to walk/cycle	21.4 (454)	20.6 (350)	1.00	-
Mixed	23.8 (589)	23 (470)	1.12	[0.79,1.59]
Not easy to walk/cycle	21.5 (1039)	21 (821)	1.09	[0.79,1.51]
**Proximity to businesses & services** ^**b**^				
†Close by	20.1 (602)	20 (465)	1.00	-
Mixed	19.8 (774)	19.7 (563)	0.93	[0.67,1.28]
Far away	25.7 (860)*	24.1 (613)	1.17	[0.86,1.60]
Likelihood ratio test v logistic regression			p = 0.31	

†Reference category.

*p < 0.05; **p < 0.01; ***p < 0.001.

^a^0 to 2 items = low score; 3 to 5 items = moderate score; 6 to 9 items = high score.

^b^Individual items were summed were summed for each scale and split into tertiles owing to the skewed distribution.

^c^Full sample N varies by each outcome due to missing data.

^d^Adjusted for all demographic, socioeconomic and environmental indicators accounting for clustering within schools.

**Table 4:** **Estimates for mean hours per week spent on physical activity on the Youth Physical Activity Question** (**Y**-**PAQ**) **by selected demographic**, **socioeconomic and environmental factors**

	**Full sample** **(N)** **Unadjusted mean** ^**c**^	**Analytic sample** **(N** **=** **1060)** **Unadjusted mean** ^**e**^	**Analytic sample** **(N** **=** **1060)** **Fully adjusted mean** **(95** **%** **CI)** ^**d**^	
**Demographic factors**				
**Gender**				
†Male	14.4 (1068)	14 (550)	14.0	[13.3,14.8]
Female	12.8 (872)***	12.6 (510)*	12.6*	[11.8,13.4]
**Ethnic group**				
†White: UK	13.6 (378)	13.4 (231)	13.5	[12.2,14.7]
White: Mixed	15 (232)	13.8 (111)	13.8	[12.0,15.5]
Asian: Indian	16.7 (71)*	17.8 (49)*	17.6*	[15.0,20.3]
Asian: Pakistani	12.8 (87)	12.2 (48)	12.2	[9.6,14.9]
Asian: Bangladeshi	12.3 (355)	12.3 (233)	12.4	[11.1,13.8]
Black: Caribbean	13 (89)	14.4 (39)	14.8	[11.9,17.8]
Black: African	13.7 (212)	13.1 (91)	13.0	[11.1,15.0]
Other	14 (498)	13.4 (258)	13.2	[12.0,14.4]
**Nativity**				
†UK Born	13.6 (1515)	13.2 (878)	13.2	[12.5,13.8]
Born overseas	13.9 (386)	13.9 (182)	14.3	[12.8,15.7]
**Borough**				
†Newham	13.8 (567)	13.7 (266)	13.5	[12.4,14.6]
Tower Hamlets	13.5 (530)	12.4 (315)	12.6	[11.5,13.7]
Barking & Dagenham	13.5 (410)	13.7 (257)	14.0	[12.8,15.1]
Hackney	14 (433)	13.9 (222)	13.5	[12.2,14.8]
**Socioeconomic factors**				
**Parental economic activity**				
†Both unemployed	13.4 (195)	13.3 (131)	14.1	[12.2,16.0]
One parent employed	13.8 (600)	13.3 (361)	13.7	[12.7,14.7]
Both parents employed	13.7 (643)	13.2 (412)	12.6	[11.6,13.7]
Lone parent employed	13.7 (137)	13.9 (80)	13.7	[11.7,15.8]
Lone parent unemployed	13.3 (119)	13.5 (70)	13.8	[11.4,16.1]
Doesn’t live with parents	16.8 (16)	14.9 (6)	13.5	[6.0,21.0]
**Family affluence** ^**a**^				
†Low	11.6 (223)	11.1 (132)	10.9	[9.2,12.5]
Moderate	13.4 (1001)*	13.1 (572)*	13.1*	[12.4,13.9]
High	15.1 (623)**	14.6 (356)***	14.6**	[13.6,15.6]
**Free school meals**				
†No meals	13.7 (1164)	13.2 (700)	13.1	[12.4,13.9]
Receives free meals	13.8 (739)	13.6 (360)	13.8	[12.6,14.9]
**Environmental Factors**				
**Neighbourhood safety** ^**b**^				
†Safe	13.4 (393)	13.3 (282)	13.4	[12.2,14.5]
Mixed	13.7 (491)	13.5 (370)	13.4	[12.5,14.4]
Not safe	13.4 (587)	13.2 (408)	13.3	[12.3,14.2]
**Neighbourhood aesthetics** ^**b**^				
†Pleasant	13.6 (343)	13.5 (271)	13.6	[12.5,14.8]
Mixed	13.3 (437)	13.2 (322)	13.2	[12.1,14.2]
Unpleasant	13.7 (671)	13.4 (467)	13.3	[12.4,14.2]
**Neighbourhood walk**-**cycleability** ^**b**^				
†Easy to walk/cycle	14.9 (277)	15 (218)	15.1	[13.9,16.3]
Mixed	13.1 (397)*	13.2 (310)*	13.2*	[12.2,14.3]
Not easy to walk/cycle	13 (687)**	12.7 (532)**	12.7**	[11.9,13.5]
**Proximity to businesses & services** ^**b**^				
†Close by	14.4 (367)	14.4 (286)	14.4	[13.3,15.5]
Mixed	12.6 (522)*	12.6 (367)*	12.6*	[11.7,13.6]
Far away	13.7 (567)	13.3 (407)	13.3	[12.4,14.2]
Likelihood ratio test v linear regression			p = 0.20	

†Reference category.

*p < 0.05; **p < 0.01; ***p < 0.001.

^a^0 to 2 items = low score; 3 to 5 items = moderate score; 6 to 9 items = high score.

^b^Individual items were summed were summed for each scale and split into tertiles owing to the skewed distribution.

^c^Full sample N varies by each outcome due to missing data.

^d^Adjusted for all demographic, socioeconomic and environmental indicators accounting for clustering within schools.

^e^Individuals reporting >75 hrs total activity per week were excluded.

**Table 5:** **Estimates for mean hours per week spent on sedentary activity on the Youth Physical Activity Question** (**Y**-**PAQ**) **by selected demographic**, **socioeconomic and environmental factors**

	**Full sample** **(N)** **Unadjusted mean** ^**c**^	**Analytic sample** **(N** **=** **1060)** **Unadjusted mean** ^**e**^	**Analytic sample** **(N** **=** **1060)** **Fully adjusted mean** **(95** **%** **CI)** ^**d**^	
**Demographic factors**				
**Gender**				
†Male	29.7 (1068)	33.2 (550)	33.1	[31.9,34.3]
Female	31.9 (872)**	35.6 (510)**	35.5**	[34.2,36.8]
**Ethnic group**				
†White: UK	31.8 (378)	34.1 (231)	33.9	[32.0,35.8]
White: Mixed	29.3 (232)	34.1 (111)	34.1	[31.4,36.8]
Asian: Indian	31.7 (71)	34.8 (49)	34.5	[30.5,38.5]
Asian: Pakistani	29.6 (87)	34.1 (48)	34.1	[30.0,38.2]
Asian: Bangladeshi	31.2 (355)	33.4 (233)	33.6	[31.5,35.6]
Black: Caribbean	32.5 (89)	36.1 (39)	35.9	[31.4,40.4]
Black: African	31 (212)	37.4 (91)	37.2	[34.3,40.2]
Other	29.8 (498)	34.1 (258)	34.0	[32.2,35.8]
**Nativity**				
†UK Born	31.4 (1515)	34.4 (878)	34.5	[33.5,35.5]
Born overseas	28.9 (386)	34.2 (182)	33.3	[31.1,35.5]
**Borough**				
†Newham	29.7 (567)	35 (266)	35.2	[33.3,37.0]
Tower Hamlets	31.7 (530)*	34.1 (315)	34.3	[32.6,36.1]
Barking & Dagenham	30.3 (410)	33.9 (257)	33.5	[31.6,35.4]
Hackney	31.3 (433)	34.3 (222)	34.0	[31.9,36.0]
**Socioeconomic factors**				
**Parental economic activity**				
†Both unemployed	31.7 (195)	34.8 (131)	34.2	[31.3,37.2]
One parent employed	29.8 (600)	33.3 (361)	33.2	[31.6,34.7]
Both parents employed	31.6 (643)	35 (412)	35.2	[33.6,36.8]
Lone parent employed	33.3 (137)	33.7 (80)	33.9	[30.7,37.1]
Lone parent unemployed	32.9 (119)	35.9 (70)	35.0	[31.4,38.6]
Doesn’t live with parents	26.4 (16)	32.5 (6)	32.2	[20.8,43.7]
**Family affluence** ^**a**^				
†Low	31.1 (223)	33.9 (132)	33.7	[31.2,36.2]
Moderate	31.4 (1001)	34.6 (572)	34.5	[33.3,35.7]
High	30.4 (623)	34 (356)	34.1	[32.5,35.6]
**Free school meals**				
†No meals	31.3 (1164)	34 (700)	33.9	[32.7,35.1]
Receives free meals	30 (739)	35 (360)	35.0	[33.2,36.8]
**Environmental Factors**				
**Neighbourhood safety** ^**b**^				
†Safe	32.8 (393)	33.5 (282)	33.4	[31.7,35.2]
Mixed	35 (491)*	35.3 (370)	35.3	[33.8,36.8]
Not safe	33 (587)	34 (408)	33.9	[32.4,35.4]
**Neighbourhood aesthetics** ^**b**^				
†Pleasant	34.1 (343)	34.2 (271)	34.2	[32.4,36.0]
Mixed	33.3 (437)	34.7 (322)	34.7	[33.1,36.3]
Unpleasant	33.3 (671)	34.1 (467)	34.0	[32.6,35.4]
**Neighbourhood walk**-**cycleability** ^**b**^				
†Easy to walk/cycle	31.8 (277)	32.9 (218)	32.8	[30.9,34.7]
Mixed	34.4 (397)*	35 (310)	34.7	[33.1,36.4]
Not easy to walk/cycle	34 (687)*	34.6 (532)	34.6	[33.3,35.8]
**Proximity to businesses & services** ^**b**^				
†Close by	32.9 (367)	33.7 (286)	33.7	[32.0,35.4]
Mixed	33.6 (522)	34.3 (367)	34.0	[32.6,35.5]
Far away	33.7 (567)	34.8 (407)	34.9	[33.4,36.3]
Likelihood ratio test v linear regression			p = <0.001	

†Reference category.

*p < 0.05; **p < 0.01.

^a^0 to 2 items = low score; 3 to 5 items = moderate score; 6 to 9 items = high score.

^b^Individual items were summed were summed for each scale and split into tertiles owing to the skewed distribution.

^c^Full sample N varies by each outcome due to missing data.

^d^Adjusted for all demographic, socioeconomic and environmental indicators accounting for clustering within schools.

^e^Individuals reporting >75 hrs total activity per week were excluded.

**Table 6:** **Prevalence estimates and odds ratios for fair**/**poor self**-**rated general health by selected demographic**, **socioeconomic and environmental factors**

	**Full sample** **(N)** **Prevalence** **%** ^**c**^	**Analytic sample** **(N** **=** **1687)** **Prevalence** **%**	**Analytic sample** **(N** **=** **1687)** **Fully adjusted odds ratio** **(95** **%** **CI)** ^**d**^	
**Demographic factors**				
**Gender**				
†Male	21.8 (1723)	20.6 (899)	1.00	-
Female	26.8 (1315)**	28.6 (788)***	1.67***	[1.32,2.12]
**Ethnic group**				
†White: UK	19 (590)	20.2 (351)	1.00	-
White: Mixed	25.5 (373)*	25.4 (185)	1.41	[0.90,2.20]
Asian: Indian	21.3 (108)	21.1 (71)	1.18	[0.61,2.27]
Asian: Pakistani	25.8 (128)	26.3 (76)	1.37	[0.75,2.51]
Asian: Bangladeshi	30.5 (501)**	29.9 (334)**	1.65*	[1.10,2.48]
Black: Caribbean	22.2 (144)	22.4 (67)	1.29	[0.67,2.51]
Black: African	24.5 (355)*	27.4 (175)*	1.81*	[1.15,2.86]
Other	23.2 (810)*	22 (428)	1.33	[0.91,1.93]
**Nativity**				
†UK Born	25 (2372)	25.4 (1372)	1.00	-
Born overseas	19.5 (614)**	19.7 (315)*	0.64**	[0.46,0.90]
**Borough**				
†Newham	25.8 (875)	28.8 (420)	1.00	-
Tower Hamlets	27.4 (793)	27.9 (476)	0.87	[0.62,1.21]
Barking & Dagenham	21.5 (657)*	21 (415)**	0.64*	[0.45,0.91]
Hackney	20.2 (713)**	18.4 (376)***	0.53**	[0.36,0.77]
**Socioeconomic factors**				
**Parental economic activity**				
†Both unemployed	28.8 (278)	29 (186)	1.00	-
One parent employed	25 (929)	24.7 (575)	0.81	[0.53,1.25]
Both parents employed	20.9 (1036)**	21.5 (671)*	0.82	[0.51,1.31]
Lone parent employed	21.6 (227)	22.9 (140)	0.85	[0.47,1.52]
Lone parent unemployed	28.1 (171)	30 (100)	1.06	[0.60,1.87]
Doesn’t live with parent	41.4 (29)	53.3 (15)	3.80	[1.24,11.66]
**Family affluence** ^**a**^				
†Low	25.6 (308)	26.3 (179)	1.00	-
Moderate	24.7 (1548)	25.5 (909)	1.05	[0.71,1.54]
High	22.6 (1048)	21.9 (599)	0.99	[0.65,1.50]
**Free school meals**				
†No meals	22.2 (1783)	22.7 (1103)	1.00	-
Receives free meals	26.6 (1197)**	27.4 (584)*	1.01	[0.75,1.37]
**Environmental Factors**				
**Neighbourhood safety** ^**b**^				
†Safe	18.6 (625)	18.7 (460)	1.00	-
Mixed	24.5 (758)**	24.6 (568)*	1.31	[0.95,1.80]
Not safe	27.7 (949)***	27.9 (659)***	1.45*	[1.04,2.01]
**Neighbourhood aesthetics** ^**b**^				
†Pleasant	20.1 (551)	20.5 (435)	1.00	-
Mixed	20.9 (681)	20.7 (513)	1.01	[0.73,1.41]
Unpleasant	28 (1056)***	29.1 (739)**	1.45*	[1.06,1.99]
**Neighbourhood walk**-**cycleability** ^**b**^				
†Easy to walk/cycle	19.8 (475)	20.1 (364)	1.00	-
Mixed	24.9 (618)*	25.1 (486)	1.32	[0.94,1.86]
Not easy to walk/cycle	25.7 (1076)*	25.7 (837)*	1.51*	[1.10,2.07]
**Proximity to businesses & services** ^**b**^				
†Close by	21 (629)	20.2 (476)	1.00	-
Mixed	23 (816)	23.4 (582)	1.13	[0.83,1.53]
Far away	27.9 (896)**	28.3 (629)**	1.51**	[1.12,2.04]
Likelihood ratio test v logistic regression			p = 0.47	

†Reference category.

*p < 0.05; **p < 0.01; ***p < 0.001.

^a^0 to 2 items = low score; 3 to 5 items = moderate score; 6 to 9 items = high score.

^b^Individual items were summed were summed for each scale and split into tertiles owing to the skewed distribution.

^c^Full sample N varies by each outcome due to missing data.

^d^Adjusted for all demographic, socioeconomic and environmental indicators accounting for clustering within schools.

**Table 7:** **Prevalence estimates and odds ratios for longstanding illness by selected demographic**, **socioeconomic and environmental factors**

	**Full sample** **(N)** **Prevalence** ^**c**^	**Analytic sample** **(N** **=** **1689)** **Prevalence** **%**	**Analytic sample** **(N** **=** **1689)** **Fully adjusted odds ratio** **(95** **%** **CI)** ^**d**^	
**Demographic factors**				
**Gender**				
†Male	42.1 (1694)	40.9 (898)	1.00	-
Female	42.6 (1310)	41 (791)	1.02	[0.84,1.25]
**Ethnic group**				
†White: UK	42.8 (584)	38.6 (352)	1.00	-
White: Mixed	48.4 (364)	50.5 (184)*	1.75**	[1.20,2.54]
Asian: Indian	40.2 (107)	36.6 (71)	1.03	[0.59,1.79]
Asian: Pakistani	48 (127)	42.1 (76)	1.18	[0.70,2.01]
Asian: Bangladeshi	39.9 (499)	38.3 (334)	1.01	[0.72,1.44]
Black: Caribbean	51.1 (139)	52.2 (67)*	1.87*	[1.09,3.22]
Black: African	31.5 (349)*	32.8 (174)	0.87	[0.58,1.31]
Other	43 (805)	42.7 (431)	1.36*	[1.00,1.86]
**Nativity**				
†UK Born	43.2 (2342)	42.1 (1372)	1.00	-
Born overseas	38 (610)*	36 (317)*	0.77	[0.58,1.02]
**Borough**				
†Newham	42.3 (863)	41.3 (421)	1.00	-
Tower Hamlets	43.1 (789)	41.7 (477)	1.02	[0.76,1.37]
Barking & Dagenham	41.9 (642)	40.8 (414)	0.98	[0.73,1.33]
Hackney	42 (710)	39.5 (377)	0.81	[0.59,1.11]
**Socioeconomic factors**				
**Parental economic activity**				
†Both unemployed	44.4 (277)	39.8 (186)	1.00	-
One parent employed	41.8 (922)	40.1 (574)	0.99	[0.67,1.45]
Both parents employed	41.3 (1024)	41.3 (671)	1.00	[0.65,1.53]
Lone parent employed	41.9 (229)	40.6 (143)	0.95	[0.58,1.58]
Lone parent unemployed	46.2 (171)	46 (100)	1.21	[0.72,2.01]
Doesn’t live with parent	41.4 (29)	40 (15)	0.92	[0.0,2.81]
**Family affluence** ^**a**^				
†Low	39.3 (303)	39.3 (178)	1.00	-
Moderate	43.2 (1534)	42.2 (912)	1.15	[0.82,1.61]
High	41 (1034)	39.4 (599)	0.99	[0.69,1.42]
**Free school meals**				
†No meals	41.5 (1755)	41.1 (1101)	1.00	-
Receives free meals	43.2 (1188)	40.6 (588)	0.89	[0.68,1.16]
**Environmental factors**				
**Neighbourhood safety** ^**b**^				
†Safe	38.2 (621)	38 (460)	1.00	-
Mixed	39.2 (755)	36.7 (570)	0.94	[0.72,1.22]
Not safe	47.3 (942)*	46.6 (659)**	1.35*	[1.03,1.78]
**Neighbourhood aesthetics** ^**b**^				
†Pleasant	37.5 (550)	36.6 (437)	1.00	-
Mixed	41.9 (677)	40.5 (511)	1.16	[0.89,1.53]
Unpleasant	44.2 (1051)**	43.7 (741)*	1.17	[0.89,1.54]
**Neighbourhood walk**-**cycleability** ^**b**^				
†Easy to walk/cycle	43 (474)	43.1 (364)	1.00	-
Mixed	40.1 (614)	39.2 (485)	0.81	[0.61,1.07]
Not easy to walk/cycle	41.1 (1074)	41 (840)	0.95	[0.74,1.23]
**Proximity to businesses & services** ^**b**^				
†Close by	41.9 (626)	39.9 (481)	1.00	-
Mixed	41.6 (806)	41.9 (580)	1.09	[0.85,1.40]
Far away	42.9 (892)	40.8 (628)	1.02	[0.79,1.31]
Likelihood ratio test v logistic regression			p = 0.39	

†Reference category.

*p < 0.05; **p < 0.01.

^a^0 to 2 items = low score; 3 to 5 items = moderate score; 6 to 9 items = high score.

^b^Individual items were summed were summed for each scale and split into tertiles owing to the skewed distribution.

^c^Full sample N varies by each outcome due to missing data.

^d^Adjusted for all demographic, socioeconomic and environmental indicators accounting for clustering within schools.

### Mental health and well-being

The complete case analysis observed that females self-reported significantly lower mental well-being than their male counterparts but there were no differences according to ethnic group or generation (Table 2). Well-being was lowest in adolescents in Newham and was significantly higher in those attending schools in Tower Hamlets and Barking & Dagenham. Overall there was a mixed relationship between well-being and socioeconomic disadvantage; there was a gradient effect whereby well-being increased significantly with increasing family affluence, but no differences were apparent according to free school meal status. However, after full adjustment, adolescents with a lone parent not in paid employment had significantly higher levels of well-being than those adolescents who had both parents outside of paid employment. However, for all environmental factors there was a statistically significant gradient effect where those perceiving the neighbourhood more positively were more likely to report higher mental well-being scores. These differences remained after full adjustment. Such patterns were broadly similar for the prevalence of depressive symptoms (Table 3). In unadjusted and adjusted models females were more likely to be at risk of depressive symptoms with no variation by socioeconomic background. However, after full adjustment only Indian adolescents were significantly less likely to report depressive symptoms than the White UK group. As observed for mental well-being, there was a significant association between negative perceptions of neighbourhood safety and aesthetics and a greater risk of depressive symptoms. This observation was also significant after full adjustment.

### Physical and sedentary activity

In unadjusted complete case models girls spent significantly fewer hours (12.6 hrs) than boys (14.0 hrs) participating in physical activity (Table 4). This was consistent with girls spending a significantly greater number of hours per week in sedentary activity (35.6 hrs) than did boys (33.2 hrs) (Table 5). Indian adolescents reported significantly higher participation in physical activity than the White UK comparison group but there were no other ethnic differences in either physical or sedentary behaviour. There was mixed evidence of a socioeconomic influence on activity. There was a significant increase in the hours spent physically active with increasing family affluence but no differences in physical and sedentary activity were observed for other socioeconomic factors. In terms of neighbourhood effects on activity, adolescents who described their neighbourhoods as more amenable to walking and cycling were significantly more likely to be physically active and less likely to be sedentary than those who described the neighbourhood as more difficult to walk or cycle. All differences remained significant in fully adjusted models. Variation in hours spent sedentary was significant at school level.

### Self-rated general health

The proportion of respondents reporting fair/(very) poor health by a range of factors is shown in Table 6. Over a quarter of girls (28.6%) reported fair/(very) poor health but the prevalence was significantly lower for boys (20.6%). Bangladeshi and Black African adolescents were significantly more likely to report poor health compared to White UK adolescents after full adjustment. There was weak evidence of a socioeconomic gradient in self-rated health. Although adolescents with both parents in employment were significantly less likely to report poor health, as were those who did not have free school meals, these differences were no longer significant in fully adjusted models. However, there was a strong and consistent association between positive perceptions of the neighbourhood and better reported general health across all three neighbourhood domains. These associations were also observed in fully adjusted models.

### Longstanding illness

Table 7 shows the prevalence estimates and factors associated with having a longstanding illness. There were no gender differences in longstanding illness. Prevalence varied widely within ethnic groups. The Black Caribbean and White Mixed groups were significantly more likely to report a longstanding illness than their White UK counterparts in unadjusted models. After adjustment the Other ethnic group was also significantly more likely to report a longstanding illness. Adolescents born overseas were significantly less likely to report having a long standing illness but this was not significant following adjustment. There were no associations between any of the socioeconomic indicators and longstanding illness. However, there was graded increase in the odds of having a longstanding illness as perceptions of neighbourhood safety and aesthetics worsened though this was no longer significant for the case of aesthetics after adjusting for covariates. No association was observed for walk/cycleability or proximity to services in the local area.

The co-occurrence of selected health outcomes after full adjustment for all covariates is described in Table 8. Fair/(very) poor general health, having a longstanding illness, lower levels of mental well-being and having depressive symptoms were all strongly associated with one another. However, there were no significant associations between the mean hours spent physically active or sedentary and all other health outcomes.

**Table 8:** **Relationships between selected health outcomes**

	**Has long term illness** ^**a**^	**Has depressive symptoms** ^**a**^	**Mean WEMWBS score** ^**b**^	**Mean hours sedentary activity** ^**b**^	**Mean hours physical activity** ^**b**^
Fair/poor general health	1.51 [1.20,1.92]***	2.17 [1.65,2.84]***	−3.78 [−4.74, 2.81]***	1.85 [−0.18,3.88]	0.25 [−1.09,1.58]
Has longstanding illness		1.59 [1.24,2.05]***	−1.38 [−2.21, −0.55]**	0.10 [−1.66,1.85]	0.16 [−0.99,1.31]
Has depressive symptoms			−7.87 [−8.83, 6.91]***	2.38 [0.20,4.56]	−0.16 [−1.56,1.27]
Mean WEMWBS score			-	0.03 [−0.01,0.06]	0.05 [−0.01,0.10]
Mean hours sedentary activity			-	-	−0.21 [−0.30,-0.12]

Models account for clustering within schools and are adjusted for gender, ethnicity, country of birth, borough, parental employment, family affluence and neighbour amenities/aesthetics/walkability/safety.

^a^Adjusted odds ratio and 95% confidence interval.

^b^Regression coefficient represents difference in score/hours.

**p <0.01; ***p <0.001.

## Discussion

This paper aimed to identify the socio-demographic and environmental determinants of a range of physical and mental health outcomes in an inner city school-based population of adolescents aged 11 to 12 years. Evidence for socioeconomic inequalities in health at this age appeared to be mixed. Though physical activity increased with family affluence and general health was worse in those receiving free school meals, there was a mixed relationship with well-being and no relationship with depressive symptoms or longstanding illness. However, the impact of the environment was much stronger and consistent across a range of neighbourhood metrics. Concurrent with previous findings across national contexts, adolescents who perceived their neighbourhoods positively had better mental health [32,33], reported better general health [34], were more likely to take part in physical exercise [35,36] and were less likely to have a longstanding illness. The association between neighbourhood perceptions and health has been repeatedly explained by the socioeconomic and demographic characteristics of the individuals. Here we controlled for a range of these confounding factors which attenuated the associations, but overall they remained significant for all outcomes. In terms of demographics there were strong gender differences with girls more likely to have poorer mental health, report poorer general health and lead a sedentary lifestyle compared to boys. Ethnic differences in reported general health in particular suggest that there are important differences between groups which must be fully understood when attempting to explain health inequalities in adolescents.

Adolescence has been described as a period of relative health equality when compared to the marked health inequalities observed in childhood and adulthood [17,37]. The Equalisation Hypothesis postulates that the health gap narrows across socioeconomic groups in early adolescence and has been described for all-cause mortality, mental health, health conditions and general health [38-41]. The hypothesis is supported by previous studies within a localised urban population of Glasgow, UK [42], with the phenomenon attributed to school and peer influences relatively outweighing home and family effects on health upon entry to adolescence. The general lack of a socioeconomic gradient in health presented here could reflect the lack of sensitivity of the Family Affluence Scale. Alternatively it is possible that equalisation may have taken place at a younger age than first reported over a decade ago. This contrasts with a recent English study based on nationally representative data purporting that equalisation occurs much later than previously proposed [43]. One explanation for the contrast between the conclusions of the local and national studies may be the influence of the urban environment on young people. It is possible that peer influences are more pervasive and family influences weaker when growing up in an urban environment. The earlier age of equalisation observed in the ORiEL study could be a consequence of adolescence occurring at an earlier age than a decade ago for all adolescents, exacerbated by the urban environment which may further promote earlier on-set adolescence.

The overall prevalence of outcomes in the ORiEL study is broadly comparable to similar studies of adolescents conducted in similar settings [16,44]. Similar aged cohorts based in South East London [45], and East London [46], have also noted that socioeconomic factors do not correlate with mental health, with exception to a limited number of ethnic minority groups. The lack of socioeconomic difference may be explained by the relative social homogeneity of the sample. In terms of self-rated general health, this outcome was associated with family wealth, albeit rather weakly. This is consistent with a comparison of material wealth and general health across 22 European countries describing similar, but declining, health inequality at this age [47]. Importantly, this study builds upon some of the earlier investigations describing a high prevalence of longstanding illness in Afro-Caribbean young adults [48]. By disaggregating this ethnic group the ORiEL study advances our knowledge by demonstrating such rates are likely to be driven by the Black Caribbean group rather than the Black African group. However there is a comparative lack of literature exploring the influence of the neighbourhood environment on adolescent health [17]. Findings presented here suggest this as useful focus for further analysis of this and other community based studies. Future studies may examine the extent to which differences in the physical environment (e.g. green or blue spaces, housing) or the social environment (e.g. crime, social cohesion) may explain differences in health with a view to providing evidence for policies aimed at reducing health inequalities via area based interventions.

The relationship between physical and mental health outcomes is consistent with previous work confirming that there is a complex and multifactorial series of pathways underpinning ill health in adolescents. However, though previous research suggests a positive association between physical activity, mental health [49,50] and general physical health [50,51], no association was observed in the ORiEL study. So while interventions aimed at increasing physical activity may well act as means of reducing obesity and its co-morbidities, they appeared unlikely to influence other health outcomes within the environmental and social context experienced by this cohort of adolescents. However, it is important to consider that these are cross-sectional observations describing associations between outcomes and their putative determinants. It is therefore possible that healthier outcomes may positively influence environmental perceptions rather than better environments leading to better health - further longitudinal examination is required to assess causality.

### Strengths and limitations

The study achieved an 87% response rate with 7% of adolescents absent on the day of the survey and 4% actively refused to participate. However, sampling weights could not be derived due to difficulties in obtaining an accurate denominator population within schools and no estimate is available of the number of adolescents educated privately. Therefore results should be generalised with caution. Nonetheless the school setting led to high levels of participant compliance suggesting that participation bias is minimal and prevalence estimates reliable. Though the young and ethnically diverse sample presented language and comprehension difficulties which could affect the validity of responses, particularly in the understanding of the mental health questions, such issues were resolved via trained fieldworkers working on an individual basis when possible. Although the WEMWBS has been validated cross-culturally and in adolescents, the range of ethnic backgrounds was limited [52] and the adolescent validation took place in a slightly older population. Despite this caveat the distribution observed for the WEMWBS scores approximates very closely to the profile observed in the nationally representative Health Survey for England 2010. This sample is well-powered to detect ethnic differences in the largest minority groups, but over 200 ethnic categories were self-reported for minor groups suggesting that the large “Other” ethnic group category is highly heterogeneous and conclusions ought to be interpreted cautiously. Due to sampling with a single school year the sample is unlikely to be confounded by age related differences in health or exposure, but there is the risk that differences in maturation may be present and possibly explain some of the significant differences in gender.

## Conclusion

This baseline study describes in detail the burden of selected health outcomes and the socio-demographic and neighbourhood correlates. This will enable valuable hypothesis generation which can be tested within the wider longitudinal study which is powered to examine causal processes. Identification of social determinants of adolescent health will facilitate the creation, implementation and evaluation of consequent interventions aimed at alleviating health inequalities at a young age which will have longer term consequences in reducing inequalities in later adult life [13]. Our findings suggest that perceptions of the physical environment, along with the social and economic characteristics of their household, are important factors in explaining patterns of health inequality experienced within this cohort.

## Abbreviations

ORiEL: Olympic Regeneration in East London
WEMWBS: Warwick Edinburgh Mental Well-Being Scale
DSM: Diagnostic statistical manual
Y-PAQ: Youth-Physical Activity Questionnaire
ALPHA: Assessing Levels of Physical Activity and Fitness
